# The albumin–bilirubin score predicts outcomes in advanced biliary tract cancer treated with durvalumab immunochemotherapy

**DOI:** 10.1093/oncolo/oyag179

**Published:** 2026-05-07

**Authors:** Ming-Yang Chen, Hao-Wei Kou, Tsung-Han Wu, Chi-Chen Lan, Jen-Shi Chen, Chia-Hsun Hsieh, Yu-Shin Hung, Wen-Chi Chou

**Affiliations:** Department of General Surgery, Chang Gung Memorial Hospital at Linkou, Taoyuan 333, Taiwan; College of Medicine, Chang Gung University, Taoyuan 333, Taiwan; Department of General Surgery, Chang Gung Memorial Hospital at Linkou, Taoyuan 333, Taiwan; College of Medicine, Chang Gung University, Taoyuan 333, Taiwan; College of Medicine, Chang Gung University, Taoyuan 333, Taiwan; Department of Hematology and Oncology, Chang Gung Memorial Hospital at Keelung, Keelung 204, Taiwan; College of Medicine, Chang Gung University, Taoyuan 333, Taiwan; Department of Hematology and Oncology, Chang Gung Memorial Hospital at Linkou, Taoyuan 333, Taiwan; College of Medicine, Chang Gung University, Taoyuan 333, Taiwan; Department of Hematology and Oncology, Chang Gung Memorial Hospital at Linkou, Taoyuan 333, Taiwan; College of Medicine, Chang Gung University, Taoyuan 333, Taiwan; Department of Hematology and Oncology, Chang Gung Memorial Hospital at Linkou, Taoyuan 333, Taiwan; Department of Hematology and Oncology, New Taipei Municipal TuCheng Hospital, New Taipei City 236, Taiwan; College of Medicine, Chang Gung University, Taoyuan 333, Taiwan; Department of Hematology and Oncology, Chang Gung Memorial Hospital at Linkou, Taoyuan 333, Taiwan; College of Medicine, Chang Gung University, Taoyuan 333, Taiwan; Department of Hematology and Oncology, Chang Gung Memorial Hospital at Linkou, Taoyuan 333, Taiwan

**Keywords:** cholangiocarcinoma, immunotherapy, prognostic model, ALBI score, real-world evidence

## Abstract

**Background:**

The albumin–bilirubin (ALBI) score objectively reflects hepatic functional reserve and is widely used as a prognostic biomarker in hepatocellular carcinoma. However, its prognostic value in patients with biliary tract carcinoma (BTC) treated with durvalumab plus gemcitabine–cisplatin (GCD) remains unclear.

**Material and Methods:**

We retrospectively analyzed 172 patients with unresectable or metastatic BTC who received first-line GCD between 2021 and 2025. Baseline ALBI scores were calculated and patients were stratified into cohort-specific tertiles (Q1-Q3). Overall survival (OS) was estimated using Kaplan–Meier method and Cox regression hazards models. Predictive performance for OS was compared between ALBI and the Eastern Cooperative Oncology Group performance status (ECOG PS) using time-dependent area under the receiver operating characteristic curves (AUC) analysis.

**Results:**

The median OS was 13.8 months (95% CI, 9.9-17.7). Survival differed significantly across ALBI tertiles: median OS was 24.0 months (95% CI, 10.3-37.8) for Q1, 14.4 months (95% CI, 9.2-19.6) for Q2, and 5.9 months (95% CI, 2.5-9.2) for Q3 (*P* < .001). In multivariate analysis, ALBI remained an independent prognostic factor for OS (Q3 vs Q1, adjusted hazard ratio 2.16; 95% CI, 1.16-4.04; *P* = .016). The ALBI score demonstrated improved prognostic discrimination (AUC 0.714 at 6 months; 0.656 at 12 months) compared with ECOG PS (AUC 0.591 and 0.551, respectively). Tumor response rates were similar across tertiles, but higher ALBI scores were associated with an increased incidence of grade ≥3 toxicities.

**Conclusion:**

The pretreatment ALBI score is a simple, objective, and independent prognostic biomarker for patients with advanced BTC receiving durvalumab-based immunochemotherapy. Incorporating ALBI into baseline assessment may help identify high-risk patients who warrant closer monitoring or individualized therapeutic strategies.

## Graphical Abstract

**Graphical Abstract:**
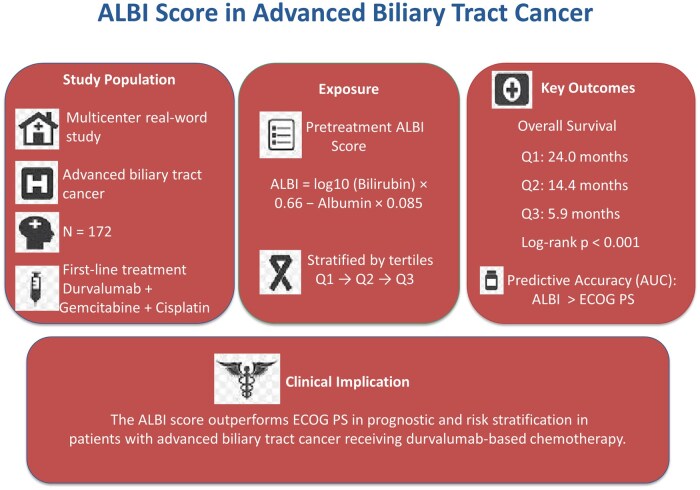

Implications for Practice The pretreatment albumin–bilirubin (ALBI) score provides a simple, objective, and readily available tool for risk stratification in patients with advanced biliary tract cancer receiving first-line durvalumab plus gemcitabine–cisplatin. In this real-world cohort, ALBI demonstrated superior prognostic discrimination compared with ECOG performance status and effectively identified a high-risk subgroup with markedly shortened survival and increased rates of severe treatment-related toxicity, particularly sepsis. Incorporation of ALBI into baseline assessment may support more individualized treatment planning. Conversely, patients with favorable ALBI scores may be better candidates for full-intensity immunochemotherapy. Routine use of ALBI could enhance clinical decision-making and improve risk-adapted management in the evolving era of immunotherapy for biliary tract cancer.

## Introduction

Biliary tract cancer (BTC) represents an aggressive malignancy characterized by a 5-year survival rate of 20%,^1^ with the majority of patients presenting with unresectable or metastatic disease.^2^ For over a decade, the combination of gemcitabine plus cisplatin has constituted the standard first-line treatment regimen, yielding a median overall survival (OS) of approximately 11 months.^3^ The phase III TOPAZ-1 trial demonstrated that the addition of the PD-L1 inhibitor durvalumab to gemcitabine–cisplatin significantly improved OS (hazard ratio [HR]: 0.80, 95% CI: 0.66-0.97), with consistent improvements observed across secondary endpoints, thereby establishing durvalumab plus gemcitabine–cisplatin (GCD) as a new first-line standard for advanced BTC.^4^^,^^5^

Despite GCD now being the preferred first-line therapy for advanced BTC, survival outcomes remain highly variable.^6^ A substantial proportion of patients experience early disease progression and limited clinical benefit, underscoring the pronounced prognostic heterogeneity within this population.^4–6^ Current prognostic assessments typically rely on individual factors such as tumor burden,^7^ Eastern Cooperative Oncology Group performance status (ECOG PS),^8^^,^^9^ the necessity for biliary drainage,^10^ and laboratory measures including bilirubin, albumin, liver enzymes, carcinoembryonic antigen (CEA), and carbohydrate antigen 19-9 (CA 19-9).^7–12^ However, these parameters are often evaluated in isolation and may not fully reflect the integrated effect of hepatic reserve on patient outcomes, a critical consideration in BTC, where liver function is frequently compromised by chronic liver disease, biliary obstruction, or prior hepatobiliary interventions.^13–15^

The albumin–bilirubin (ALBI) score, originally developed to provide an objective, evidence-based assessment of liver functional reserve in hepatocellular carcinoma, was calculated from serum albumin and bilirubin concentrations using a standardized formula.^16^ This continuous laboratory-based index objectively reflects the synthetic and excretory capacities of the liver and has demonstrated prognostic utility in diverse chronic liver diseases, including viral hepatitis,^17^ cirrhosis,^18^ metabolic dysfunction-associated fatty liver disease,^19^ and hepatocellular carcinoma.^20^^,^^21^ Compared with the traditional Child–Pugh classification, ALBI eliminates subjective components and has demonstrated superior discriminatory ability for survival prediction in several settings.^22^

Although the ALBI score has been widely validated as a prognostic biomarker across hepatobiliary malignancies and increasingly investigated in biliary tract cancer populations, including surgical and chemotherapy-treated settings,^23^^,^^24^ its clinical utility in patients receiving contemporary immunochemotherapy remains incompletely defined. In the context of immunotherapy, liver function is particularly relevant, as immune checkpoint inhibitors may induce immune-related hepatitis, and systemic inflammation, frequently reflected by hypoalbuminemia, has been shown to modulate the anti-tumor immune response by promoting an immunosuppressive tumor microenvironment and impairing effective T-cell–mediated immunity.^12^ Therefore, compromised hepatic function, as captured by a higher ALBI score, may reflect both reduced treatment tolerance and a less favorable immunological milieu for effective anti-tumor activity. This study aims to investigate the independent prognostic value of the pretreatment ALBI score in a real-world cohort of patients with advanced BTC receiving first-line durvalumab-based immunochemotherapy. Our hypothesis is that a higher ALBI score, signifying poorer liver function, will be independently associated with worse overall and progression-free survival.

## Methods

### Study design and participants

**Figure 1.**
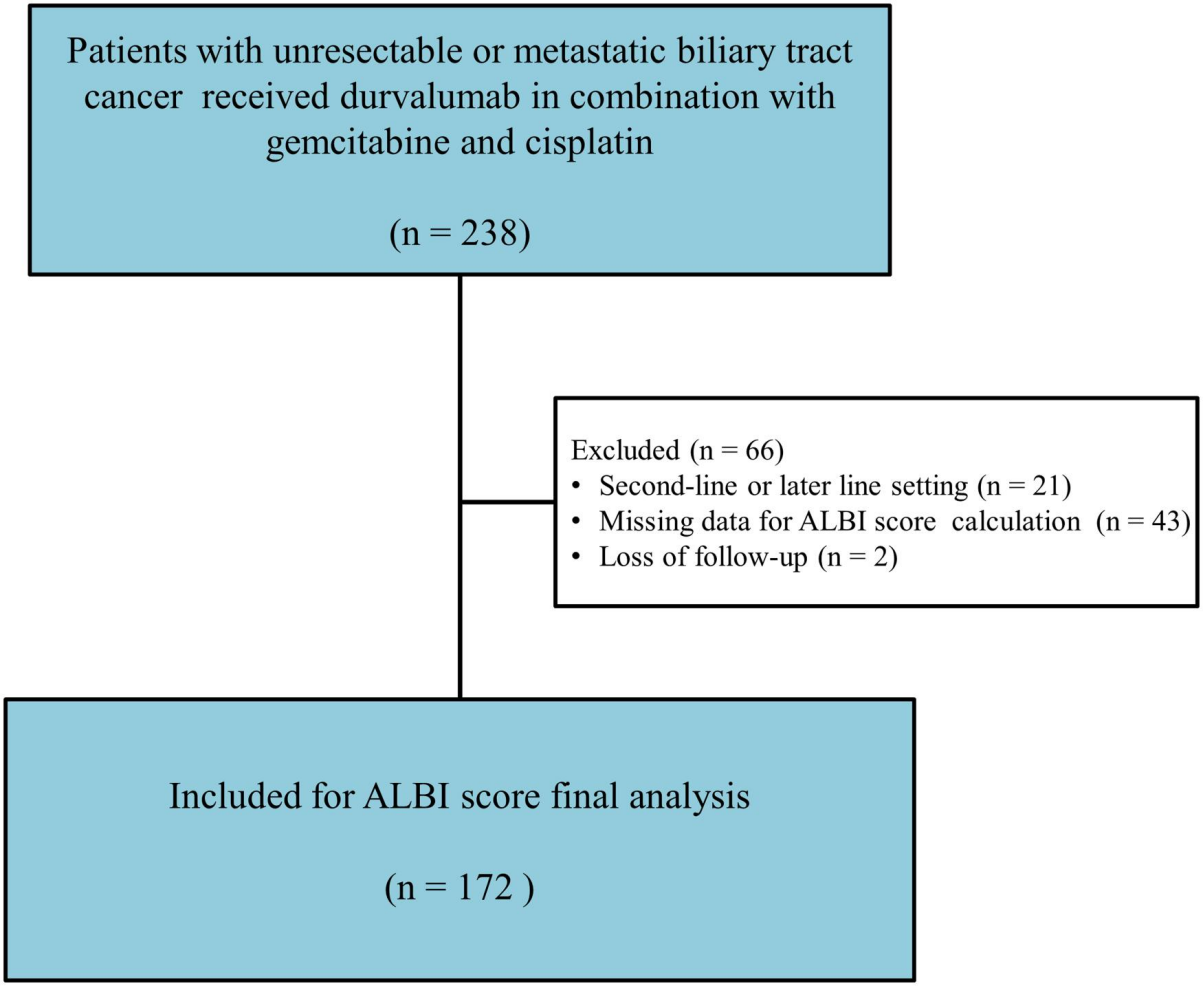
Study flowchart.

This retrospective, multicenter observational study was conducted at 3 academic medical institutions in Taiwan. We identified a cohort of consecutive patients with histologically confirmed, unresectable, recurrent, or metastatic BTC who received first-line GCD between August 2021 and March 2025 as recorded in the institutional databases. Recurrent disease was defined as radiologically or pathologically confirmed relapse following prior curative-intent surgery without prior systemic therapy for advanced disease, thereby meeting the definition of first-line treatment. Eligibility criteria encompassed histological confirmation of BTC, age >20 years, completion of at least one full cycle of GCD therapy, and availability of baseline serum albumin and bilirubin levels for calculation of the ALBI score prior to the initiation of treatment. Patients were excluded if they received durvalumab as second-line or later therapy or did not receive durvalumab as part of first-line treatment, had missing clinical or laboratory data necessary for ALBI score determination, or were lost to follow-up. The Institutional Review Board of each participating center approved the study protocol (IRB No. 202501211B0). The requirement for written informed consent was waived due to the retrospective nature of the analysis. Figure 1 illustrates the study flowchart.

### Treatment protocol

In accordance with the TOPAZ-1 study protocol,^4^ patients were administered durvalumab (1500 mg intravenously on day 1 of each 21-day cycle) plus gemcitabine (1000 mg/m^2^) and cisplatin (25 mg/m^2^) on days 1 and 8 for up to 8 cycles, followed by durvalumab maintenance (1500 mg every 4 weeks) until disease progression, unacceptable toxicity, withdrawal of consent, or death.

### ALBI score calculation

The ALBI score was calculated using the formula:

ALBI score = (log10 bilirubin total [µmol/L]) ×0.66 – (albumin [g/L] ×0.085).^16^ Patients were stratified into cohort-specific tertiles (Q1–Q3) based on the distribution of ALBI scores. The corresponding cutoff values were as follows: Q1, ≤ −2.76; Q2, −2.76 ∼ −2.28; and Q3, ≥ −2.28. For reference, conventional ALBI grades (Q1, ≤ −2.60; Q2, −2.60 ∼ −1.39; and Q3, ≥ −1.39) were also explored descriptively.^16^

### Outcome evaluation

OS was defined as the time from the initiation of GCD treatment to death from any cause or to the last follow-up (censored on June 30, 2025). PFS was defined as the time from the initiation of GCD treatment to the first documented disease progression, death, or the last follow-up. Imaging evaluations were conducted every 8-12 weeks, per institutional practice.

Tumor responses and adverse events were assessed according to the Response Evaluation Criteria in Solid Tumors version 1.1^25^ and the Common Terminology Criteria for Adverse Events (CTCAE) version 5.0,^26^ respectively. Sepsis was defined based on CTCAE criteria and corroborated by clinical documentation, including physician diagnosis and/or microbiological evidence when available.

### Statistical analysis

Categorical variables are summarized as counts and percentages, and continuous variables as medians with ranges. Baseline characteristics were compared across ALBI tertiles using the chi-square test or Fisher’s exact test for categorical variables and the Kruskal–Wallis test for continuous variables.

Survival curves for each ALBI tertile were constructed using the Kaplan–Meier method and subsequently compared using Cox regression analysis. The prognostic significance of the ALBI tertiles on OS and PFS was evaluated using univariate and multivariate Cox proportional hazards regression models with HRs and 95% CIs reported. The proportional hazards assumption was assessed using Schoenfeld residuals, and no significant violations were observed. Variables with *P* < .05 in the univariate analysis were entered into multivariate models. Albumin and bilirubin were excluded from the multivariate model for OS as they are components of the ALBI score. The discriminatory capacity of the ALBI tertile classification was evaluated using time-dependent receiver operating characteristic curves (AUC) and the Akaike information criterion (AIC) were generated for OS prediction at 6- and 12 months. All statistical tests were 2-sided, with *P* < .05 considered statistically significant. Analyses were conducted using SPSS software (version 23.0; IBM Corp.) and R software (version 4.3.0; R Foundation for Statistical Computing).

## Results

### Patient characteristics

A total of 172 patients were included, of whom 102 (59.3%) were male (Table 1). The median age was 64 (range, 35-88) years, and 28.5% of the patients were aged >70 years. Most patients had an ECOG PS of 1 (69.8%) and metastatic disease at the time of diagnosis (70.3%). The intrahepatic bile duct was the most common primary tumor site (65.1%) and 28.5% of patients had undergone prior surgical resection. Biliary drainage was performed in 40.1% patients.

**Table 1.** Baseline characteristics, *n* (% or range).

Characteristics	Entire cohort, *n* = 172	ALBI Q1, *n* = 58	ALBI Q2, *n* = 58	ALBI Q3, *n* = 56	*P* value
**Sex**					.53
**Male**	102 (59.3)	35 (60.3)	37 (63.8)	30 (53.6)	
**Female**	70 (40.7)	23 (39.7)	21 (36.2)	26 (46.4)	
**Age, median (range)**	64 (35-88)	60 (35-83)	65 (44-87)	68 (40-88)	.006
**≤70 years**	123 (71.5)	49 (84.5)	38 (65.5)	36 (64.3)	.03
**>70 years**	49 (28.5)	9 (15.5)	20 (34.5)	20 (35.7)	
**ECOG performance**					.22
**0**	35 (20.3)	16 (27.6)	12 (20.7)	7 (12.5)	
**1**	120 (69.8)	40 (69.0)	40 (71.4)	40 (71.4)	
**2**	12 (7.0)	2 (3.4)	4 (6.9)	6 (10.7)	
**3**	5 (2.9)	0	2 (3.4)	3 (5.4)	
**Primary tumor site**					.11
**Intrahepatic**	112 (65.1)	44 (75.9)	34 (58.6)	34 (60.7)	
**Extrahepatic**	60 (34.9)	14 (24.1)	24 (41.4)	22 (39.9)	
**Stage**					.59
**Locally advanced**	51 (29.7)	15 (25.9)	20 (34.5)	16 (28.6)	
**Metastatic**	121 (70.3)	43 (74.1)	38 (65.5)	40 (71.4)	
**Surgery**					.09
**Yes**	49 (28.5)	22 (37.9)	16 (27.6)	11 (19.6)	
**No**	123 (71.5)	36 (62.1)	42 (72.4)	45 (80.4)	
**Biliary drainage**					<.001
**Yes**	69 (40.1)	10 (17.2)	28 (48.3)	31 (55.4)	
**No**	103 (59.9)	48 (82.8)	30 (51.7)	25 (46.6)	
**Albumin**					<.001
**Normal value**	124 (72.1)	58 (100)	51 (87.9)	15 (26.8)	
**Decreased value**	48 (27.9)	0	7 (12.1)	41 (73.2)	
**Bilirubin**					<.001
**Normal value**	130 (75.6)	56 (96.6)	47 (81.0)	27 (48.2)	
**Increased value**	42 (24.4)	2 (3.4)	11 (19.0)	29 (51.8)	
**AST**					.001
**Normal value**	115 (66.9)	46 (79.3)	42 (72.4)	27 (48.2)	
**Increased value**	57 (33.1)	12 (20.7)	16 (27.6)	29 (51.8)	
**ALT**					.02
**Normal value**	109 (63.4)	42 (72.4)	40 (69.0)	27 (48.2)	
**Increased value**	63 (36.6)	16 (27.6)	18 (31.0)	29 (51.8)	
**GGT**					.30
**Normal value**	25 (14.5)	11 (19.0)	9 (15.5)	5 (8.9)	
**Increased value**	147 (85.5)	47 (81.0)	49 (84.5)	51 (91.1)	
**CA 19-9**					.45
**Normal value**	52 (30.2)	21 (36.2)	15 (25.9)	16 (28.6)	
**Increased value**	120 (69.8)	37 (63.8)	43 (74.1)	40 (71.4)	
**CEA**					.04
**Normal value**	88 (51.2)	37 (63.8)	28 (48.3)	23 (41.1)	
**Increased value**	84 (48.8)	21 (36.2)	30 (51.7)	33 (58.9)	

Abbreviations: ALT, alanine transaminase; AST, aspartate transaminase; CA 19-9, carbohydrate antigen 19-9; CEA, carcinoembryonic antigen; ECOG, Eastern Cooperative Oncology Group; GGT, gamma-glutamyl transferase.

When stratified by ALBI tertile, patients in Q3 were significantly older (median, 68 years) than those in Q1 (median, 60 years) and Q2 (median, 65 years; *P *= .006). A higher proportion of Q3 patients required biliary drainage (55.4% vs 17.2% in Q1 and 48.3% in Q2; *P* < .001) and had decreased albumin levels (73.2% vs 0% and 12.1%, respectively; *P* < .001) or elevated total bilirubin levels (51.8% vs 3.4% and 19.0%, respectively; *P* < .001). Abnormal liver function test results, including elevated aspartate aminotransferase (AST; *P* = .001) and alanine aminotransferase (ALT; *P* = .02) levels, were more common in Q3. CEA elevation was significantly more frequent in Q3 than in Q1 (*P* = .04), whereas no significant differences were observed across the tertiles for sex, ECOG PS, tumor stage, prior surgery, g-glutamyl-transferase (GGT), carbohydrate antigen 19-9 (CA19-9), or primary tumor site.

### Survival outcomes

**Figure 2.**
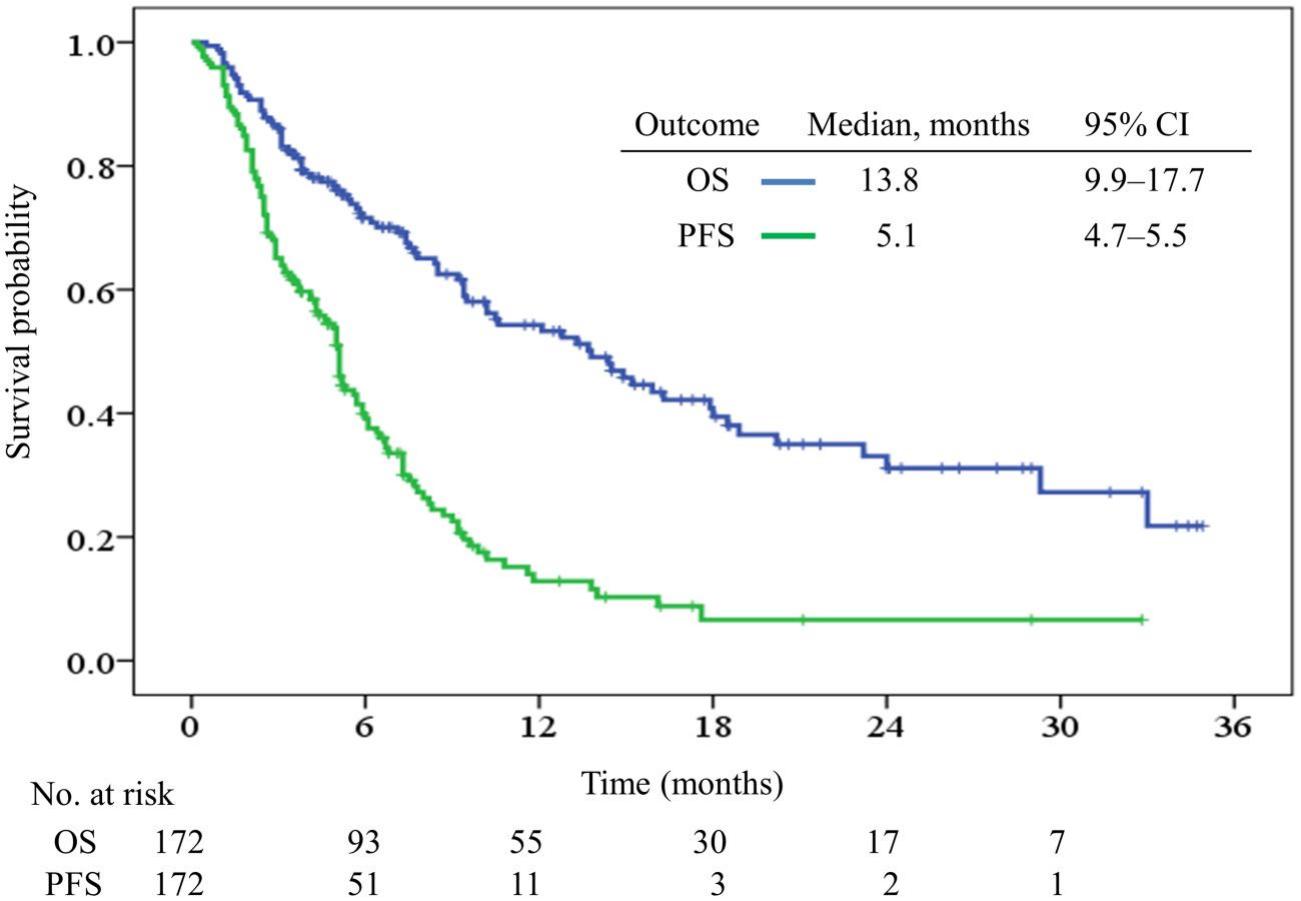
Kaplan–Meier curves for overall survival (OS) and progression-free survival (PFS) in the entire patient cohort.

With a median follow-up of 15.0 (range, 2.6-34.9) months, 86 patients (50.0%) had died by the conclusion of the study period. In the overall cohort, the median OS was 13.8 months (95% CI, 9.9-17.7), and the median PFS was 5.1 months (95% CI, 4.7-5.5) (Figure 2).

**Figure 3.**
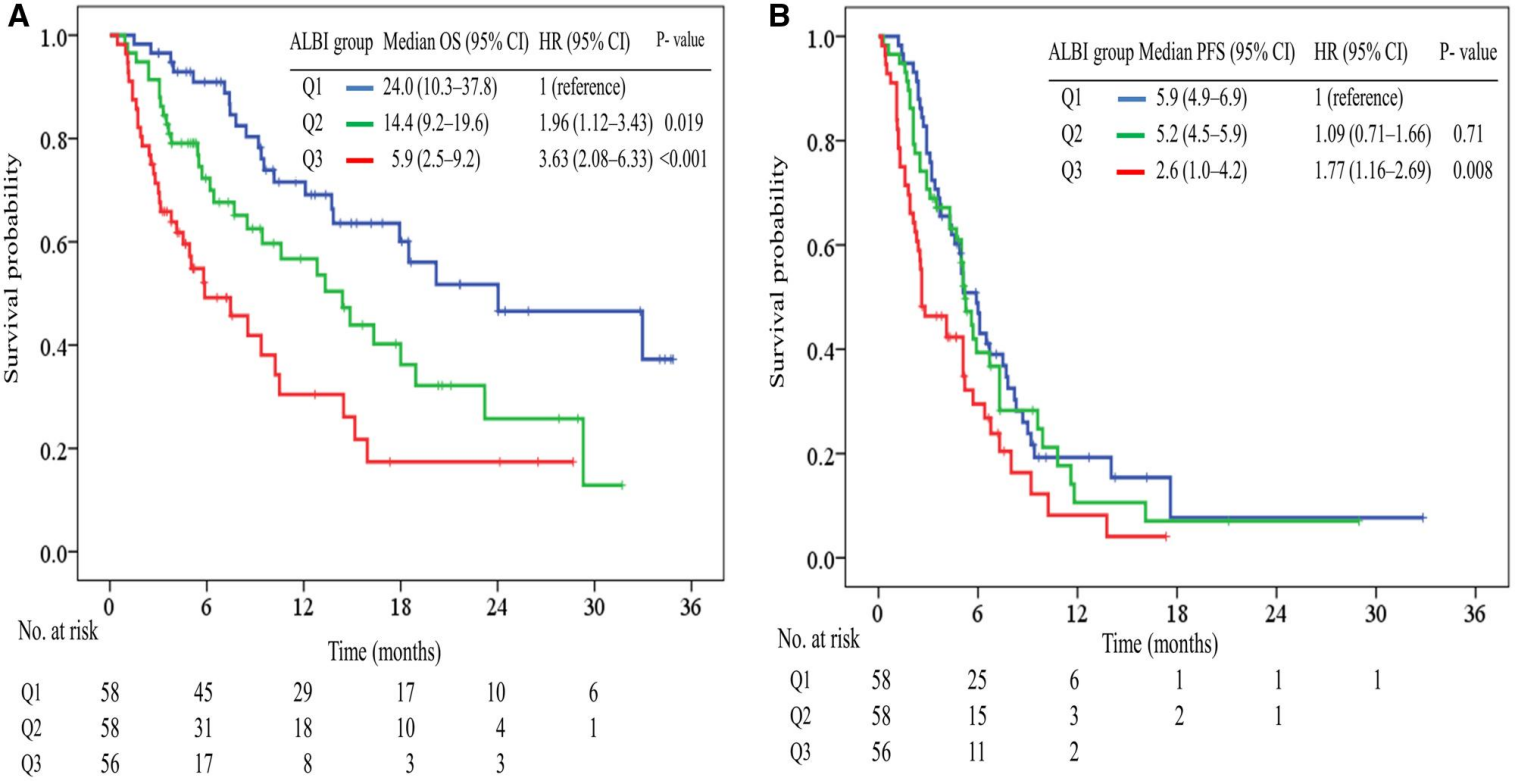
Kaplan–Meier curves for overall survival (A) and progression-free survival (B), stratified by albumin–bilirubin (ALBI) score tertiles. Patients were categorized into 3 groups based on liver function: Q1, Q2, and Q3.

OS differed significantly among the 3 ALBI tertiles (log-rank *P* < .001) (Figure 3A). The median OS was 24.0 months (95% CI, 10.3-37.8) for ALBI Q1, 14.4 months (95% CI, 9.2-19.6) for ALBI Q2, and 5.9 months (95% CI, 2.5-9.2) for ALBI Q3. Compared with Q1, the risk of death was significantly higher in Q2 (HR, 1.96; 95% CI, 1.12-3.43; *P* = .019) and Q3 (HR, 3.63; 95% CI, 2.08-6.33; *P* < .001).

PFS exhibited significant variation among the 3 ALBI tertiles (log-rank *P *< .001) (Figure 3B). The median PFS was 5.9 months (95% CI, 4.9-6.9) for ALBI Q1, 5.2 months (95% CI, 4.5-5.9) for ALBI Q2, and 2.6 months (95% CI, 1.0-4.2) for ALBI Q3. Compared with Q1, the risk of disease progression was not significantly different in Q2 (HR, 1.09; 95% CI, 0.71-1.66; *P* = .71) but was significantly higher in Q3 (HR, 1.77; 95% CI, 1.16-2.69; *P* = .008).

To enhance external applicability, we additionally performed exploratory survival analyses using the conventional ALBI grade classification (Grade 1: ≤ −2.60; Grade 2: > −2.60 to ≤ −1.39; Grade 3: > −1.39). Consistent with the primary tertile-based analysis, OS differed significantly across conventional ALBI grades (log-rank *P* < .001). Median OS was 18.5 months (95% CI, 15.2-21.8) for Grade 1, 8.5 months (95% CI, 6.3-10.8) for Grade 2, and 10.2 months (95% CI, 0.9-19.5) for Grade 3 (Figure S1a, see online supplementary material for a color version of this figure). Compared with Grade 1, the risk of death was significantly higher in Grade 2 (HR, 2.10; 95% CI, 1.33-3.31; *P* = .001) and Grade 3 (HR, 3.32; 95% CI, 1.52-7.25; *P* = .003). PFS showed a numerically shorter trend with worsening conventional ALBI grade, although the differences were less pronounced than for OS (Figure S1b, see online supplementary material for a color version of this figure). Median PFS was 5.3 months (95% CI, 4.4-6.2) for Grade 1, 5.0 months (95% CI, 3.5-6.5) for Grade 2, and 5.2 months (95% CI, 0-11.3) for Grade 3. Compared with Grade 1, the HR for progression was 1.38 (95% CI, 0.97-1.97; *P* = .07) for Grade 2 and 1.77 (95% CI, 0.57-2.51; *P* = .63) for Grade 3.

### Prognostic factors for overall survival

Table S1 presents the findings of univariate and multivariate analyses of OS. In the univariate analysis, age ≥70 years (HR, 2.24; 95% CI, 1.44-3.49; *P* < .001), ECOG PS ≥2 (HR, 4.68; 95% CI, 2.52-8.69; *P* < .001), metastatic disease (HR, 2.54; 95% CI, 1.45-4.45; *P* = .001), decreased albumin (HR, 3.04; 95% CI, 1.95-4.73; *P* < .001), elevated AST (HR, 2.07; 95% CI, 1.34-3.18; *P* = .001), elevated GGT (HR, 3.12; 95% CI, 1.84-5.28; *P* < .001), and higher ALBI tertiles (Q2 vs Q1: HR, 1.96; 95% CI, 1.12-3.43; *P* = .019; Q3 vs Q1: HR, 3.63; 95% CI, 2.08-6.33; *P* < .001) were significantly associated with shorter OS.

In multivariate analysis, independent predictors of poorer OS included age ≥70 years (adjusted HR, 1.91; 95% CI, 1.19-3.07; *P* = .007), ECOG PS ≥2 (adjusted HR, 2.35; 95% CI, 1.23-4.50; *P* = .010), metastatic disease (adjusted HR, 2.69; 95% CI, 1.50-4.81; *P* = .001), elevated GGT (adjusted HR, 2.13; 95% CI, 1.17-3.85; *P* = .013), and ALBI Q3 vs Q1 (adjusted HR, 2.16; 95% CI, 1.16-4.04; *P* = .016).

### Predictive accuracy for survival probability

**Figure 4.**
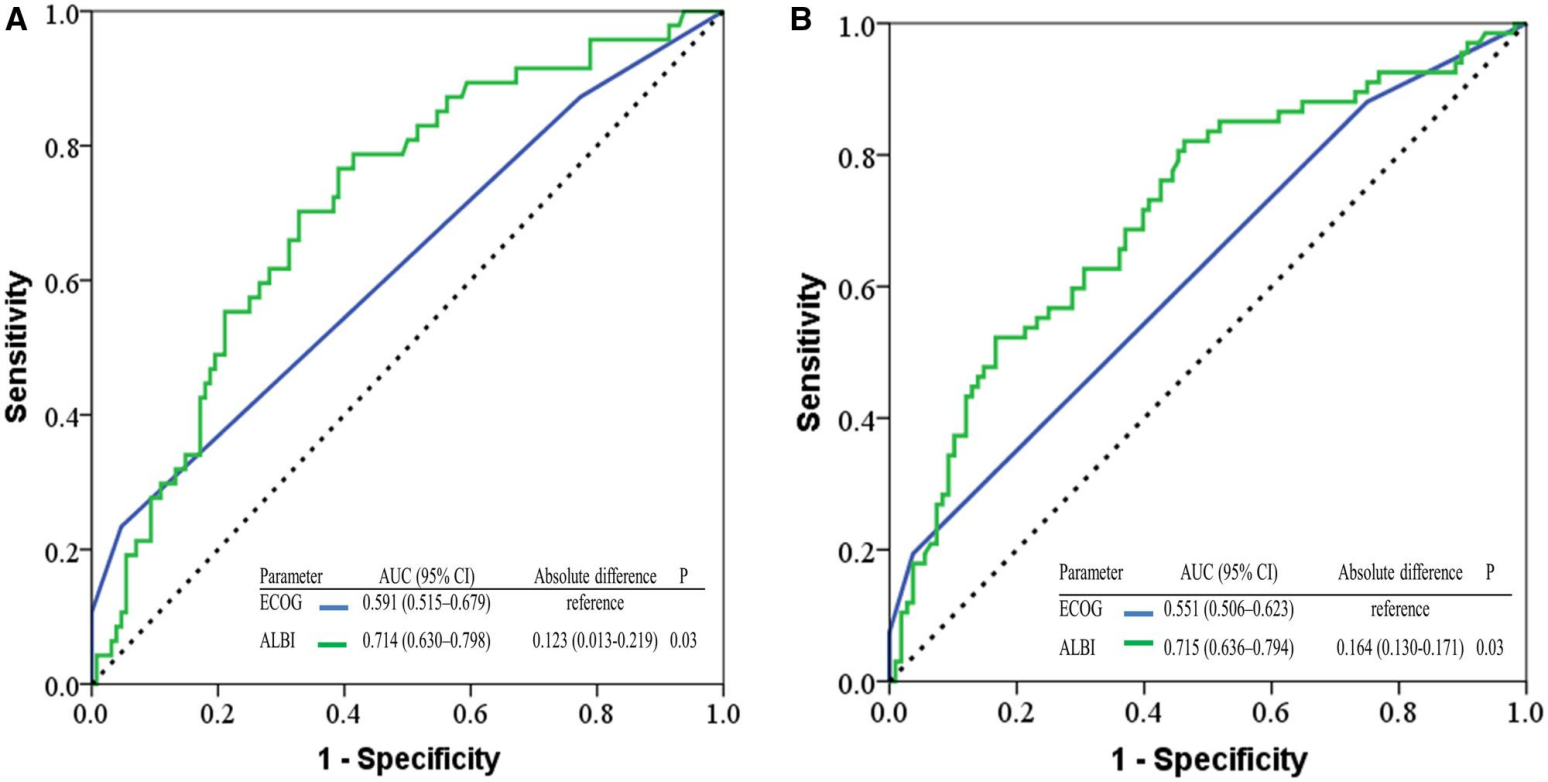
Time-dependent receiver operating characteristic curves for predicting 6-month (A) and 12-month (B) overall survival. The analysis compares the predictive performance of the albumin–bilirubin (ALBI) score and the Eastern Cooperative Oncology Group performance status (ECOG PS).

The ROC curve (AUC) for predicting the 6-month and 12-month survival probability is shown in Figure 4. For predicting 6-month survival probability (Figure 4A), the AUC was 0.591 (95% CI, 0.515-0.679) for ECOG PS and 0.714 (95% CI, 0.630-0.797) for the ALBI score, analyzed as a continuous variable. The absolute AUC difference between ALBI and ECOG PS was 0.123 (95% CI, 0.013-0.219), which was statistically significant (*P* = .03). The AIC values favored the ALBI model over ECOG performance status (181.9 vs 189.7), indicating a better model fit.

Similarly, for 12-month survival prediction (Figure 4B), the ALBI score showed improved discrimination compared with ECOG performance status (AUC 0.715 vs 0.551), with a statistically significant difference (absolute difference 0.164, 95% CI: 0.130-0.171; *P* = .03).

The corresponding AIC values were 223.2 for the ECOG PS and 220.1 for ALBI, again favoring the ALBI in terms of model fit.

### Tumor response

In the entire cohort, the best tumor response distribution was as follows: complete response (CR) in 3 patients (1.7%), partial response (PR) in 40 patients (23.3%), stable disease (SD) in 77 patients (44.8%), and progressive disease (PD) in 52 patients (30.2%) (Table 2). The distribution of best tumor responses differed significantly among the tertiles (*P* = .040); however, no clear monotonic trend was observed across increasing ALBI tertiles. In ALBI Q1, the CR, PR, SD, and PD rates were 1.7%, 27.6%, 44.8%, and 25.9%, respectively. In ALBI Q2, the corresponding rates were 3.4%, 27.6%, 43.1%, and 25.9%, respectively, whereas in ALBI Q3, they were 0%, 14.3%, 46.4%, and 39.9%, respectively.

**Table 2.** Summary of treatment response, *n* (%).

Parameter, *n* (%)	Enteric cohort, *n* = 172	ABLI Q1, *n* = 58	ABLI Q2, *n* = 58	ABLI Q3, *n* = 56	*P*
**Best tumor response**					.04
**Complete response**	3 (1.7%)	1 (1.7%)	2 (3.4%)	0	
**Partial response**	40 (23.3%)	16 (27.6%)	16 (27.6%)	8 (14.3%)	
**Stable disease**	77 (44.8%)	26 (44.8%)	25 (43.1%)	26 (46.4%)	
**Progressive disease**	52 (30.2%)	15 (25.9%)	15 (25.9%)	22 (39.9%)	
**Objective response rate**	43 (25.0%)	17 (29.3%)	18 (31.0%)	8 (14.3%)	.07
**[95% CI]**	[18.7%-32.2%]	[18.1%-42.7%]	[19.5%-44.5%]	[6.4%-26.2%]	
**Disease control rate**	120 (69.8%)	43 (74.1%)	43 (74.1%)	34 (60.7%)	.12
**[95% CI]**	[62.3%-76.5%]	[61.0%-84.7%]	[61.0%-84.7%]	[46.8%-73.5%]	

The objective response rate (ORR) for the entire cohort was 25.0%, with higher rates observed in Q1 (29.3%) and Q2 (31.0%) than in Q3 (14.3%); however, the difference was not statistically significant (*P* = .07). The disease control rate (DCR = CR + PR + SD) was 69.8% overall, with rates of 74.1% in Q1 and Q2 and 60.7% in Q3 (*P* = .12).

### Safety profile

Table 3 summarizes the grade 3 or 4 adverse events in the study cohort. Overall, 117 patients (68.0%) experienced at least one grade ≥3 toxicity, with a significantly higher incidence in ALBI Q2 (77.6%) and Q3 (76.8%) compared with Q1 (50.0%; *P* = .001). The most frequent hematological toxicities were anemia (26.7%), thrombocytopenia (20.3%), neutropenia (18.6%), and leukopenia (16.3%). Anemia and thrombocytopenia occurred more often in ALBI Q2 and Q3 than in ALBI Q1 (*P* = .001 and *P* = .024, respectively).

**Table 3.** Safety profiles (*n* = 172).

Grade 3 or 4 adverse events, *n* (%)	Entire cohort, *n* = 172	ALBI Q1, *n* = 58	ALBI Q2, *n* = 58	ALBI Q3, *n* = 56	*P*
**Any toxicity**	117 (68.0%)	29 (50.0%)	45 (77.6%)	43 (76.8%)	.001
**Anemia**	46 (26.7%)	5 (8.6%)	22 (37.9%)	19 (33.9%)	.001
**Thrombocytopenia**	35 (20.3%)	5 (8.6%)	15 (25.9%)	15 (26.8%)	.02
**Neutropenia**	32 (18.6%)	14 (24.1%)	12 (20.7%)	6 (10.7%)	.16
**Leukopenia**	28 (16.3%)	10 (17.2%)	12 (20.7%)	6 (10.7%)	.34
**Neutropenic fever**	4 (2.3%)	0	1 (1.7%)	3 (5.4%)	.15
**Hyponatremia**	10 (5.8%)	2 (3.4%)	3 (5.2%)	5 (8.9%)	.44
**Hypokalemia**	25 (14.5%)	3 (5.2%)	9 (15.5%)	13 (23.2%)	.02
**AST elevation**	6 (3.5%)	3 (5.2%)	3 (5.2%)	0	.22
**ALT elevation**	8 (4.7%)	4 (6.9%)	4 (6.9%)	0	.13
**Creatinine elevation**	1 (0.6%)	0	0	1 (1.8%)	.35
**Fatigue**	5 (2.9%)	1 (1.7%)	2 (3.4%)	2 (3.6%)	.80
**Anorexia**	7 (4.1%)	2 (3.4%)	3 (5.2%)	2 (3.6%)	.87
**Emesis**	1 (0.6%)	0	0	1 (1.8%)	.35
**Skin rash**	4 (2.3%)	2 (3.4%)	1 (1.7%)	1 (1.8%)	.78
**Sepsis**	64 (37.2%)	8 (13.8%)	26 (44.8%)	30 (53.6%)	<.001
**Colitis (≥ grade 2)**	2 (1.2%)	0	0	2 (3.6%)	.12
**irAE (≥ grade 2)**	9 (5.2%)	1 (1.7%)	4 (6.9%)	4 (7.1%)	.34

Abbreviations: ALT, alanine transaminase; AST, aspartate transaminase; irAE, immune-related adverse event.

Among the non-hematological toxicities, hypokalemia (14.5%) and sepsis (37.2%) were the most common, with significantly higher rates in patients with higher ALBI scores (*P* = .023 and *P* < .001, respectively). Other grade ≥3 events, including hyponatremia, transaminase elevations, fatigue, anorexia, skin rash, and immune-related adverse events, were less frequent (<10% each) and did not significantly differ among ALBI tertiles. Colitis (≥ grade 2) occurred in only 1.2% of patients.

## Discussion

The addition of durvalumab to standard gemcitabine and cisplatin chemotherapy, based on the landmark TOPAZ-1 trial, has established a new standard of care for patients with advanced BTC.^4^ Despite this significant therapeutic advancement, the survival benefit is heterogeneous, underscoring the critical need for effective, easy-to-use biomarkers to accurately stratify patient risk and personalize treatment intensity.^6^ Our study confirms that the pretreatment ALBI score is a clinically relevant, independent prognostic biomarker for patients with advanced BTC receiving first-line durvalumab-based immunochemotherapy. A clear, step-wise reduction in both OS and PFS was demonstrated across increasing ALBI scores (Q1-Q3), establishing its meaningful association with outcomes. The ALBI score demonstrated a more consistent and stepwise association with OS than with PFS. This pattern suggests that baseline hepatic reserve may have a greater influence on long-term outcomes, including treatment tolerance and post-progression survival, rather than on early tumor control alone. Our findings align with the growing body of literature supporting the ALBI score in other gastrointestinal malignancies, such as hepatocellular carcinoma.^20^^,^^21^ To our knowledge, this is among the larger multicenter real-world cohorts evaluating ALBI in GCD-treated advanced BTC and uniquely links ALBI stratification to severe toxicity, particularly sepsis, while quantifying discrimination relative to ECOG performance status.

To enhance the external applicability of our findings, we additionally evaluated survival outcomes using the conventional ALBI grade classification. Although our primary analysis used cohort-specific tertiles to ensure balanced risk groups and avoid assumptions regarding predefined cutoffs, the conventional grading system demonstrated generally consistent prognostic patterns, particularly for OS. Patients with higher conventional ALBI grades experienced shorter survival and increased mortality risk, supporting the broader reproducibility of the association between impaired hepatic reserve and adverse outcomes in this treatment setting. The separation in PFS was less pronounced, which may reflect the multifactorial determinants of early tumor progression and the limited number of patients classified as ALBI Grade 3. Collectively, these findings suggest that the prognostic relevance of the ALBI score is preserved across different classification approaches and reinforce its potential clinical utility as a practical biomarker in patients receiving first-line GCD.

Compared to the durvalumab arm of TOPAZ-1,^4^ our cohort exhibited notable differences in baseline characteristics. Although the median age was identical in both groups (64 years), the proportion of patients with an ECOG PS of 0 was substantially lower in our cohort (20.3% vs 50.7%), and a lower proportion had metastatic disease at diagnosis (70.3% vs 88.9%). These differences suggest that our patient population was functionally impaired, reflecting the reality of routine clinical practice. The median OS in our study was 13.8 months, slightly longer than the 12.9 months reported for TOPAZ-1, whereas the median PFS was 5.1 months compared with 7.2 months in TOPAZ-1.^4^ The shorter PFS observed in our cohort may partly reflect less standardized disease assessment in real-world settings, where imaging is performed less frequently and without centralized radiologic review, leading to earlier or inconsistent classification of progression compared with the rigorously monitored trial setting. Nevertheless, the survival outcomes in our cohort were comparable to those reported from real-world experiences, with OS ranging from 10 to 16 months and PFS ranging from 5.0 to 7.6 months.^9^^,^^27–30^

The strong prognostic capability of the ALBI score in this treatment setting is likely multifactorial, reflecting a confluence of hepatic functional reserve, systemic inflammation, and treatment tolerance.^11^ BTC frequently involves the liver, making liver functional reserve a critical determinant of patient health.^10^^,^^15^ A compromised liver, signified by a high ALBI score (low albumin, high bilirubin), directly limits the host’s capacity to metabolize cytotoxic agents like cisplatin and gemcitabine.^31–33^ This can lead to increased drug toxicity, necessitating dose reductions or treatment delays, which ultimately diminish the efficacy of the chemo-immunotherapy backbone. The ALBI score serves as a simple, objective surrogate for the host’s systemic inflammatory and nutritional status.^16^ Low serum albumin is a hallmark of cancer-related cachexia and chronic systemic inflammation, known to promote an immunosuppressive tumor microenvironment.^12^^,^^34^ This elevated systemic inflammation often correlates with higher levels of immune-suppressive cells,^34^ which can actively counteract the anti-tumor effects of PD-L1 blockade by durvalumab.^35^^,^^36^ Thus, a high ALBI score reflects both poor liver function and a less favorable immunological landscape, making patients less likely to derive durable benefit from the immune checkpoint inhibitor component of the treatment.

A key strength of the ALBI score lies in its objectivity and ease of quantification. In contrast to subjective assessments such as ECOG performance status, which may be influenced by inter-observer variability and patient-related factors,^37^ the ALBI score is derived from readily available laboratory parameters and provides a standardized assessment of hepatic functional reserve.^16^ While ECOG performance status remains an important prognostic factor,^10^ our findings suggest that the ALBI score captures a complementary dimension of host physiological vulnerability. In this study, the ALBI score demonstrated improved prognostic discrimination for 6- and 12-month survival compared with ECOG performance status, as reflected by higher AUC and lower AIC values; however, the overall predictive performance remained moderate. These findings support the role of ALBI as a practical and objective biomarker that may contribute to risk stratification when integrated with other clinical factors, rather than as a standalone prognostic model.

Although ECOG performance status was used as a comparator in this study due to its widespread clinical use, it represents a relatively global and subjective assessment of patient condition. More comprehensive prognostic tools, including liver function–based indices such as the Model for End-Stage Liver Disease score^38^ or disease-specific models such as the MEGNA score,^39^ may provide additional prognostic granularity. However, these models require variables that were not uniformly available in our retrospective dataset or are less routinely applied in standard oncology practice. Therefore, the ALBI score should be interpreted as a practical and complementary biomarker, and future prospective studies are warranted to directly compare its performance with established prognostic models in patients receiving contemporary immunochemotherapy.

The pretreatment ALBI score provides a simple, objective, and readily available tool for risk stratification in patients with advanced biliary tract cancer receiving first-line GCD. Although ALBI is not a novel biomarker and has been consistently associated with outcomes across hepatobiliary malignancies,^16^ our findings offer context-specific validation in a contemporary real-world immunochemotherapy cohort. Higher ALBI scores were associated with shorter survival and increased rates of severe treatment-related toxicity, particularly sepsis, highlighting its ability to capture host vulnerability beyond traditional performance status measures. These findings support the use of ALBI as a prognostic biomarker to inform risk-adapted clinical management, including closer monitoring, optimization of supportive care, and shared decision-making.^40^^,^^41^ However, given the observational nature of this study, the ALBI score should not be used to guide treatment selection or dosing strategies without prospective validation.

Beyond predicting survival, our analysis revealed a highly significant and clinically impactful association between baseline ALBI score and treatment-related toxicity, specifically the incidence of sepsis (*P* < .001), with patients in the highest tertile (ALBI Q3) experiencing markedly higher rates of sepsis (53.6%). This finding likely reflects multiple, interrelated mechanisms. Impaired hepatic reserve, as indicated by a higher ALBI score, may compromise the liver’s capacity to detoxify metabolic byproducts, synthesize key immune mediators, and maintain adequate systemic albumin levels,^11^^,^^12^ thereby contributing to an immunocompromised state and increased susceptibility to severe infections, especially in the context of myelosuppressive chemotherapy.^13^ In addition, patients with higher ALBI scores more frequently required biliary drainage and had underlying biliary obstruction, both of which are established risk factors for biliary tract infections. Together, these factors underscore the complex interplay between hepatic dysfunction, biliary interventions, and infection risk, which may collectively contribute to the high incidence of sepsis observed in this high-risk subgroup.

Our study possesses several strengths, including its multicenter design, relatively large sample size, and detailed capture of both the efficacy and safety outcomes in a real-world setting, thereby enhancing the applicability of our findings to routine clinical practice.^42^ The primary limitations of this study include its retrospective design and the inherent selection biases of a real-world cohort, although the use of multivariate analysis helps mitigate these risks. Furthermore, the ALBI score is a dynamic marker, and this study utilized only a single, pretreatment measurement. Future prospective investigations are warranted to assess the prognostic impact of changes in the ALBI score during the course of durvalumab-based immunochemotherapy, which may provide an even more sensitive tool for treatment response monitoring. Finally, although this study was conducted across multiple institutions, the overall sample size remains modest, reflecting the relatively recent adoption of durvalumab-based immunochemotherapy in routine practice. Larger studies, ideally based on national registries or broader multi-institutional collaborations, are warranted to validate these findings and enhance their generalizability.

## Conclusion

The pretreatment ALBI score is a simple and objective biomarker associated with survival outcomes in patients with advanced BTC receiving first-line durvalumab-based immunochemotherapy. This score is readily calculated using standard laboratory tests and effectively stratifies patients into distinct risk groups, offering a practical and clinically relevant tool to personalize therapeutic strategies and improve prognostication in the current era of BTC care.

## Supplementary Material

oyag179_Supplementary_Data

## Data Availability

The datasets used and/or analyzed in the current study are available from the corresponding author upon reasonable request.
