# Supplementary material for: The albumin–bilirubin score predicts outcomes in advanced biliary tract cancer treated with durvalumab immunochemotherapy
Source: Oncologist. 2026 May 7;31(6):oyag179. doi: 10.1093/oncolo/oyag179 (PMC13200058; doi:10.1093/oncolo/oyag179)

Supplementary Table. Univariate and multivariate analyses for overall survival

| Characteristics | Univariate | |  | Multivariate | |
| --- | --- | --- | --- | --- | --- |
|  | HR ((95% CI) | P value |  | Adjusted HR ((95% CI) | p |
| Gender  Male  Female | 1  0.78 (0.51–1.20) | 0.26 |  |  |  |
| Age  < 70  ≥ 70 | 1  2.24 (1.44–3.49) | <0.001 |  | 1  1.91 (1.19–3.07) | 0.007 |
| ECOG performance  0-1  ≥ 2 | 1  4.68 (2.52–8.69) | <0.001 |  | 1  2.35 (1.23–4.50) | 0.010 |
| Primary site  Intrahepatic  Extrahepatic | 1  0.90 (0.58–1.42) | 0.66 |  |  |  |
| Stage  Locally  Meta | 1  2.54 (1.45–4.45) | 0.001 |  | 1  2.69 (1.50–4.81) | 0.001 |
| Surgery  Yes  no | 1  1.24 (0.88–2.01) | 0.39 |  |  |  |
| Biliary drainage  No  Yes | 1  1.37 (0.89-2.10) | 0.15 |  |  |  |
| Albumin  Normal value  Decreased value | 1  3.04 (1.95–4.73) | <0.001 |  | 1  2.58 (1.99–3.23) | 0.001 |
| Bilirubin  Normal value  Increased value | 1  1.15 (0.68–1.95) | 0.59 |  |  |  |
| AST  Normal value  Increased value | 1  2.07 (1.34–3.18) | 0.001 |  | 1  1.46 (0.91–2.33) | 0.12 |
| ALT  Normal value  Increased value | 1  1.38 (0.89–2.12) | 0.14 |  |  |  |
| GGT  Normal value  Increased value | 1  3.12 (1.84–5.28) | <0.001 |  | 1  2.13 (1.17–3.85) | 0.013 |
| CA199  Normal value  Increased value | 1  1.45 (0.92–2.29) | 0.11 |  |  |  |
| CEA  Normal value  Increased value | 1  1.06 (0.69–1.63) | 0.80 |  |  |  |
| ALBI tertile  Q1  Q2  Q3 | 1  1.96 (1.12-3.43)  3.63 (2.08-6.33) | 0.019  <0.001 |  | 1  1.55 (0.84–2.77)  2.16 (1.16–4.04) | 0.13  0.016 |

ECOG PS, Eastern Cooperative Oncology Group; ALT, alanine transaminase; AST, aspartate transaminase; GGT, gamma glutamyl transferase; CA 19-9: carbohydrate antigen 19-9; CEA, carcinoembryonic antigen; NLR, neutrophil-to-lymphocyte ratio.

Supplementary Figure 1. Kaplan–Meier curves for overall survival (a) and progression-free survival (b), stratified by conventional albumin-bilirubin (ALBI) grades (Q1, ≤ -2.60; Q2, -2.60 ~ -1.39; and Q3, ≥ -1.39)). Patients were categorized into three groups based on liver function: Q1 (blue line), Q2 (green line), and Q3 (red line).


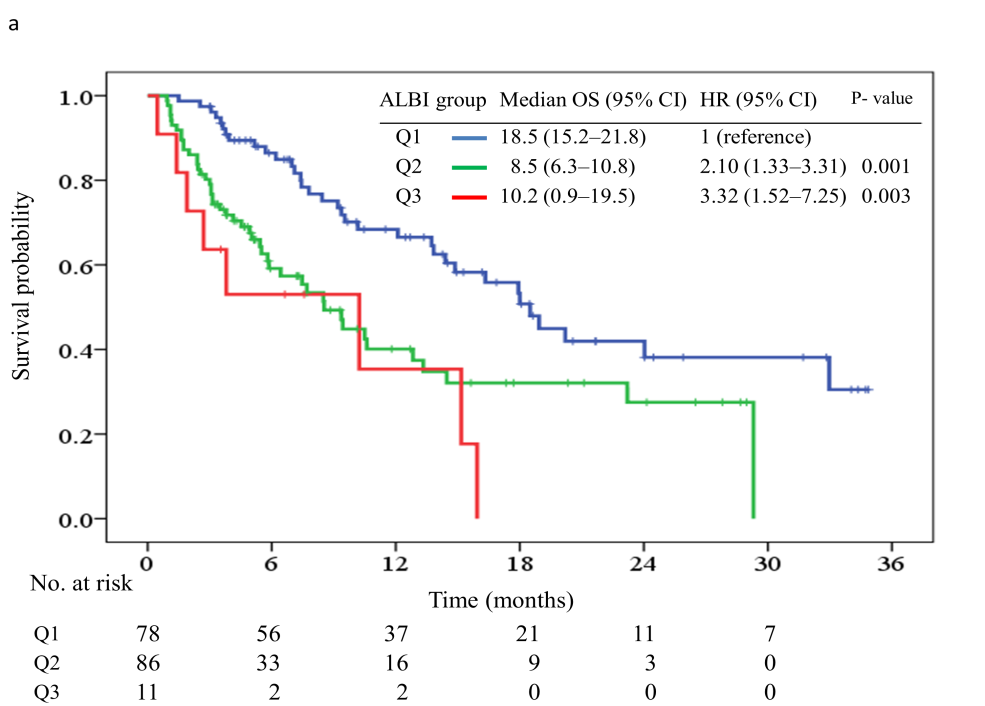

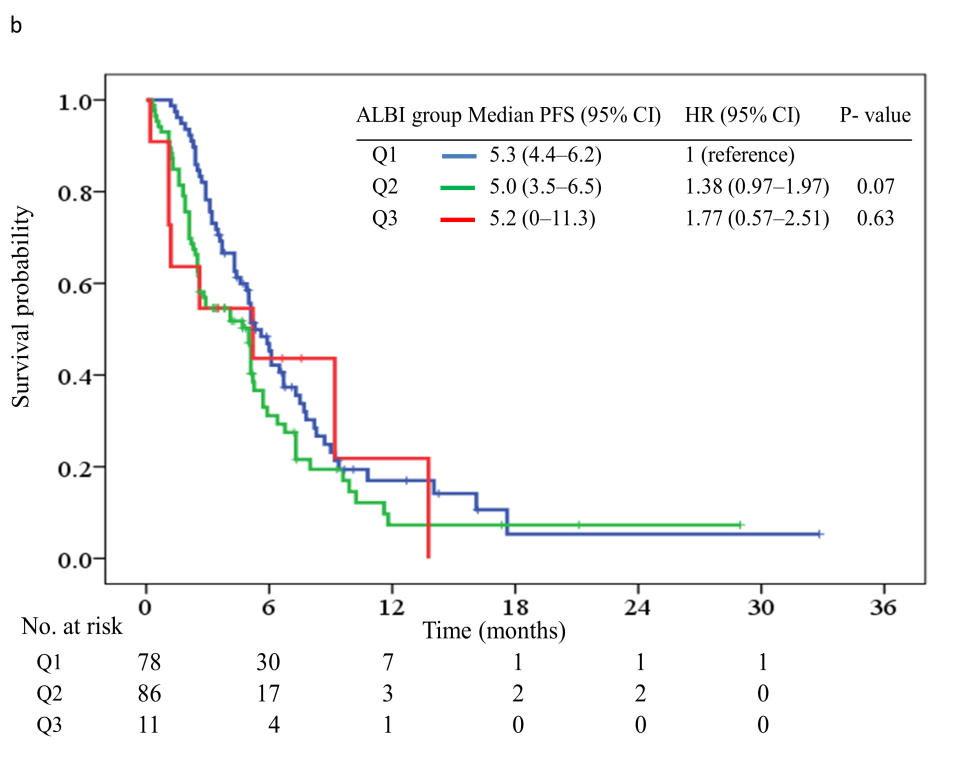

Supplement: oyag179_Supplementary_Data [file oyag179_supplementary_data.docx]
